# Kaposi sarcoma in a men-who-have-sex-with-men patient without human immunodeficiency virus who was treated with upadacitinib for ulcerative colitis

**DOI:** 10.1016/j.jdcr.2024.06.005

**Published:** 2024-06-18

**Authors:** Jacob T. Tribble, Mckinzie Johnson, Yeanna Moon, Anand Rajpara, Jacob Whitsitt

**Affiliations:** a Department of Dermatology, University of Missouri – Kansas City School of Medicine, Kansas City, Missouri; b Department of Pathology, University of Colorado School of Medicine, Aurora, Colorado; c UCHealth Medical Group, Denver, Colorado

**Keywords:** herpesvirus, immunosuppression, JAK inhibitor, Kaposi sarcoma, MSM, ulcerative colitis

## Introduction

Kaposi sarcoma (KS) is a rare neoplasm of lymphatic endothelium-derived cells infected with human herpesvirus 8 (HHV-8).^1^ There are 4 variant forms of KS: classic, AIDS-related, endemic (African), and iatrogenic.^1^ Recent literature has noted an emerging fifth variant of KS seen in men-who-have-sex-with-men (MSM) but who are HIV-seronegative.^2^ With the growing use of immune modulating medications such as Janus kinase (JAK) inhibitors for a range of inflammatory diseases, there have been an increasing number of reported iatrogenic KS cases associated with these medications.^3^ We report a case of KS in a patient who is MSM with a history of severe ulcerative colitis (UC) treated with upadacitinib, highlighting both the potential risk of malignancy associated with JAK inhibitors and risk of KS in MSM patients without HIV.

## Case report

**Fig 1:**
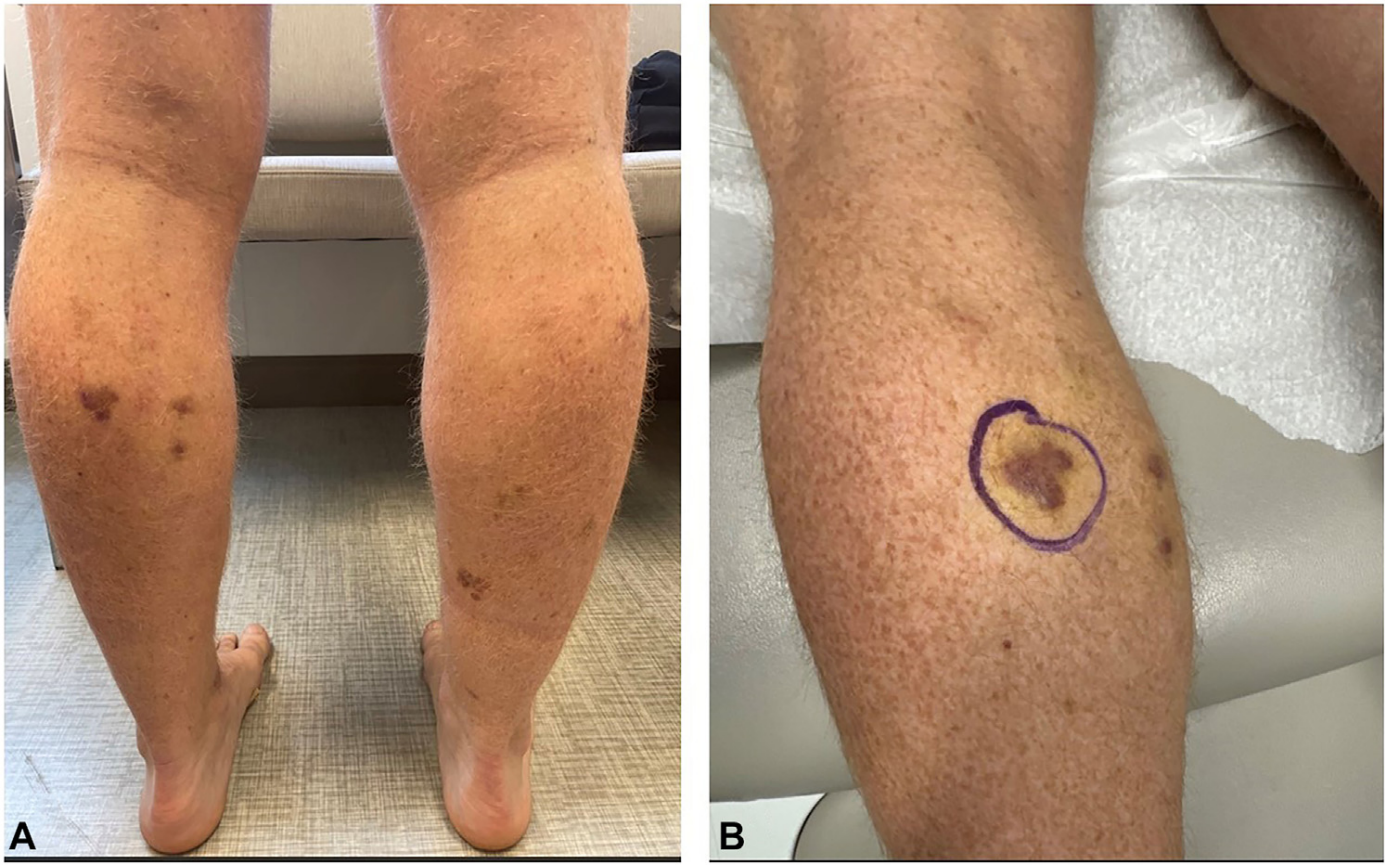
Initial presentation of Kaposi sarcoma. **A,** Patient’s bilateral calves. **B,** Patient’s left calf and site of skin biopsy.

**Fig 2:**
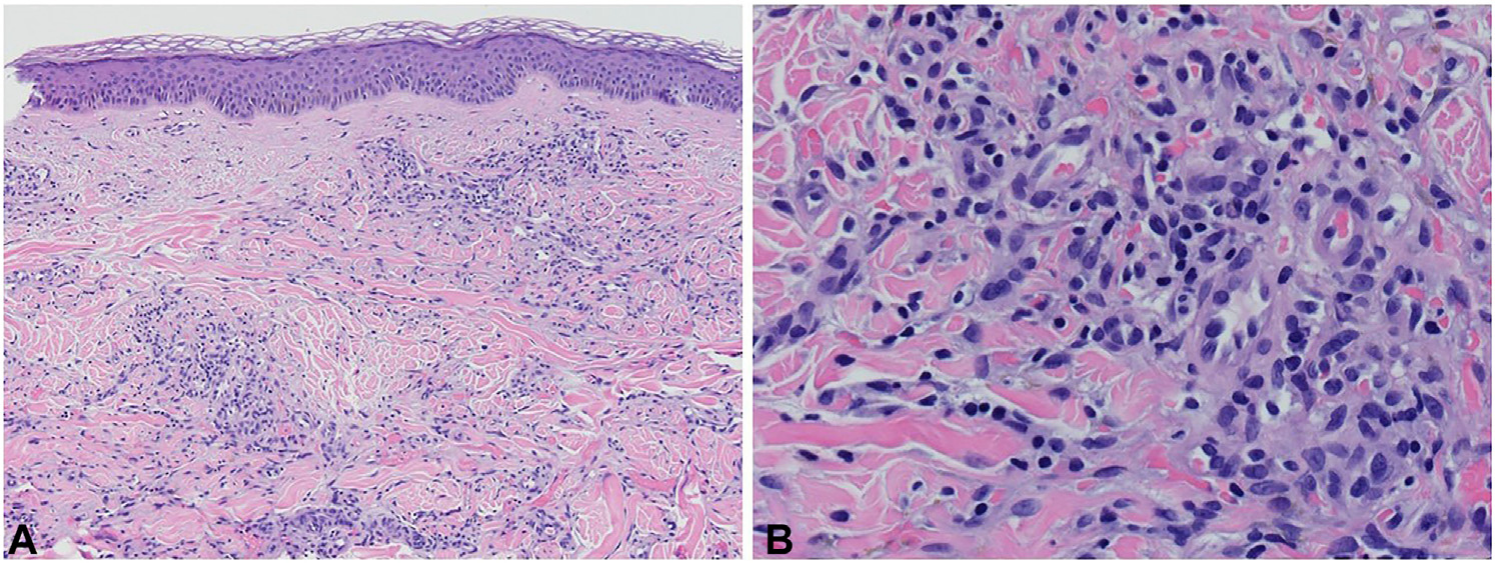
Histopathology of the lesion on the patient’s left calf using a hematoxylin and eosin stain showing cellular proliferation composed of spindled to histiocytoid cells infiltrating between collagen bundles and entrapping native vascular structures, with lesional cells forming slit-like poorly formed vascular channels. (Original magnifications: **A,** ×10; **B,** ×40.)

**Fig 3:**
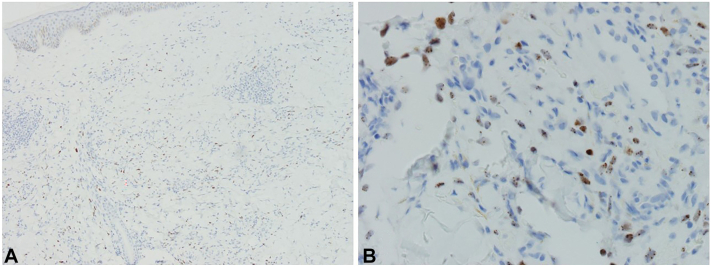
Immunohistochemistry from a send-out stain for HHV-8 performed at NeoGenomics—labeling nuclei of lesional cells. (Original magnifications: **A,** ×10; **B,** ×40.)

A 45-year-old man presented to the dermatology clinic with a 6-month history of slowly progressive violaceous plaques on his bilateral lower extremities. Medical history was significant for preexposure prophylaxis use for HIV prevention because he has male sexual partners and severe UC that failed to improve with adalimumab therapy. He was switched to upadacitinib 15 mg daily for treatment resistant UC 1 year before his presentation to the dermatology clinic. Six months before he presented to the clinic, his dose of upadacitinib was increased to 30 mg daily. After his dose was increased, over the next 6 months he developed slowly progressive asymptomatic violaceous papules and plaques, first involving his bilateral ankles, with later involvement of his bilateral calves. Physical examination on the day of presentation to the dermatology clinic was congruent with these findings (Fig 1). Skin biopsy was taken of the lesion on the patient’s left calf, and histopathology showed the proliferation of spindled cells infiltrating collagen bundles and native vascular structures throughout the dermis (Fig 2). Immunohistochemistry showed neoplastic cells diffusely positive for HHV-8, confirming the diagnosis of KS (Fig 3). HIV-1/2 antigen/antibody testing 2 weeks after the skin biopsy was collected was negative. Upper and lower gastrointestinal endoscopy along with a computed tomography of chest, abdomen, and pelvis with contrast were all negative for visceral KS involvement. At this point, medical oncology was consulted and believed the best course of action would be to discontinue the upadacitinib and monitor the KS for progression while switching him to vedolizumab for management of his UC. At the time of this case report, the patient had canceled his appointments and was no longer able to be monitored.

## Discussion

Most commonly, iatrogenic KS is seen in solid organ transplant recipients.^1^ However, as JAK inhibitors have received approval for a wide range of diseases throughout the past decade, there have been a growing number of KS cases related to their use.^3^ JAK inhibitors act by targeting JAKs, which are tyrosine kinases that have a pivotal role in cellular signal transduction for a wide range of systems.^4^ JAK inhibitors have quickly revolutionized the management of autoimmune and inflammatory dermatologic disease. Because of their immunosuppressive properties, there are a range of adverse effects seen with these medications.^4^

JAK inhibitors have been demonstrated to increase the risk of herpes zoster, with a recent systematic review looking at 47 randomized controlled trials showing a 3 times higher odds of shingles in those treated with upadacitinib compared with those without treatment.^5^ Basic science studies have demonstrated the crucial role of JAK pathways in the proliferation of CD4^+^ lymphocytes for optimal antiviral functions.^6^ It is possible that downregulation of these pathways by JAK inhibitors could cause varicella-zoster virus reactivation and lead to increased herpes zoster incidence.^6^ Theoretically, the same could be said about HHV-8 reactivation leading to KS development in patients treated with JAK inhibitors. This report contributes to a growing number of iatrogenic KS reports. Although there are too few cases to be assessed systematically, there are at least 7 other reported cases of KS associated with JAK inhibitors to date.^3^ Interestingly, in 3 of these KS cases, regression was seen with discontinuation of the JAK inhibitor without any other therapies, indicating a causal role of JAK inhibitors in the pathogenesis.^3^

Aside from KS, JAK inhibitors have been associated with an increased risk of malignancy. A recent meta-analysis found an increased risk of malignancies in patients using JAK inhibitors compared with those using tumor necrosis factor-alfa inhibitors, but no difference when compared with placebo or those using methotrexate.^7^ Because of the relatively small number of cancer events and short amount of follow-up time for placebos, this meta-analyses was limited in examining more detailed analyses on the risk of individual cancer subtypes.^7^ As more data becomes available, future research should examining the risk of KS and other virus induced cancers in those using JAK inhibitors, as we know the JAK pathway plays a critical role in the immune system.

A novel form of KS appears to be arising in the population of MSM without HIV, of which our case is another example. Epidemiologic studies in the United States have shown increased prevalence of HHV-8 in MSM ranging from 30% to 70% compared with the general population at 4%.^8^^,^^9^ Although this is increased prevalence of HHV-8 is known, it is difficult to quantify the risk MSM without HIV have in KS development compared with the general population because cancer registries do not routinely collect information on sexual orientation and gender identity.

This case highlights the potential risk of KS among those using JAK inhibitors and those who are MSM without HIV. Future studies should aim to assess the risk of KS development in patients using JAK inhibitors and collect information on sexual orientation and gender identity. Additional reports may inform practitioners on the utility of screening for HHV-8 among MSM before starting immunomodulatory medicines. Lastly, physician’s prescribing JAK inhibitors should be aware of and monitor for potential KS development along with other malignancies and opportunistic infections.

## Conflicts of interest

None disclosed.
